# First Report of a Methicillin-Resistant, High-Level Mupirocin-Resistant *Staphylococcus argenteus*

**DOI:** 10.3389/fcimb.2022.860163

**Published:** 2022-03-15

**Authors:** Adebayo Osagie Shittu, Franziska Layer-Nicolaou, Birgit Strommenger, Minh-Thu Nguyen, Stefan Bletz, Alexander Mellmann, Frieder Schaumburg

**Affiliations:** ^1^Department of Microbiology, Obafemi Awolowo University, Ile-Ife, Nigeria; ^2^Institute of Medical Microbiology, University Hospital Münster, Münster, Germany; ^3^National Reference Centre (NRC) for Staphylococci and Enterococci, Division of Nosocomial Pathogens and Antibiotic Resistances, Department of Infectious Diseases, Robert Koch Institute, Wernigerode Branch, Wernigerode, Germany; ^4^Institute for Hygiene, University Hospital Münster, Münster, Germany

**Keywords:** identification, methicillin-resistant *Staphylococcus argenteus*, high-level mupirocin resistance, plasmid, whole-genome sequencing (WGS)

## Abstract

We describe the identification of a methicillin-resistant, high-level mupirocin-resistant *Staphylococcus argenteus*. The isolate (1801221) was characterized as t6675-ST2250-SCC*mec*IVc, and whole-genome sequencing revealed that the isolate possessed two plasmids. One plasmid (34,870 bp), designated p1_1801221 with *rep23*, harboured the mupirocin resistance (*mupA*) gene. The second plasmid (20,644 bp), assigned as p2_1801221 with *rep5a* and *rep16*, carried the resistance determinants for penicillin (*blaZ*) and cadmium (*cadD*). Phylogenetic analysis revealed that the isolate clustered with the European ST2250 lineage. The overall high similarity of both plasmids in *S. argenteus* with published DNA sequences of *Staphylococcus aureus* plasmids strongly suggests an interspecies transfer. The pathogenic potential, community and nosocomial spread, and acquisition of antibiotic resistance gene determinants, including the *mupA* gene by *S. argenteus*, highlight its clinical significance and the need for its correct identification.

## Introduction

*Staphylococcus argenteus* and *S. schweitzeri*, with *S. roterodami* and *S. singaporensis*, are recently designated species and assigned to the *Staphylococcus aureus*-related complex (Tong et al., 2015; Chew et al., 2021; Schutte et al., 2021). *S. argenteus* and *S. aureus* demonstrate similar reactions to key biochemical tests for phenotypic characterization with identical 16S rRNA gene sequences (Tong et al., 2015). Hence, it is difficult to distinguish these two species by routine diagnostic methods (Kaden et al., 2018; Tunsjø et al., 2018). Various tools have been developed to differentiate *S. argenteus* from the *S. aureus*-related complex (Becker et al., 2019). They include Matrix-assisted laser-desorption ionization time-of-flight mass spectrometry (MALDI-TOF MS) (Chen et al., 2018a) and PCR detection of the nonribosomal peptide synthetase (NRPS) gene (Zhang et al., 2016). *S. argenteus* was previously considered less virulent than *S. aureus* due to the lack of the carotenoid pigment, staphyloxanthin (Holt et al., 2011), which impairs oxidative stress and neutrophil killing (Liu et al., 2005). However, *S. argenteus* possesses similar *S. aureus* virulence determinants (Zhang et al., 2017), including the gene encoding Panton-Valentine leukocidin (PVL) (Chantratita et al., 2016).

There are increasing reports of *S. argenteus* infections worldwide (Chantratita et al., 2016; Alhussein et al., 2020; Diot et al., 2020; Hao et al., 2020; Mitsutake et al., 2020; Eshaghi et al., 2021). *S. argenteus* isolates are generally penicillin-resistant (*blaZ*-positive) (Becker et al., 2019), but in Europe, methicillin-resistant (MR)-*S. argenteus* (>10 isolates) have been identified in Denmark (Hansen et al., 2017), Netherlands (Bank et al., 2021) and Sweden (Hallbäck et al., 2018; Giske et al., 2019). Also, a recent study (Goswami et al., 2021) revealed that of the *S. argenteus* genomes deposited in the public databases, 20% were *mecA*-positive. Becker et al. (2019), in a position paper on the *S. aureus*-related complex, suggested adopting infection prevention and control measures similar to methicillin-resistant *S. aureus* (MRSA) guidelines on a laboratory report of MR-*S. argenteus* in human infections. The application of mupirocin ointment on the mucous membrane (e.g., anterior nares) is an important strategy for decolonizing patients and healthcare personnel with MRSA (Patel et al., 2009). However, the emergence of resistance is associated with unrestricted policies and antibiotic use for long periods in healthcare settings (Hetem and Bonten, 2013). Two levels of *S. aureus* resistance to mupirocin have been elucidated, i.e., low-level and high-level resistance (HmupR) attributed to mutation and the acquisition of plasmids, respectively (Patel et al., 2009). Whereas the prevalence of MRSA with HmupR is 5.9%, 8.0%, and 12.1% in the Americas, Europe, and Asia, respectively (Dadashi et al., 2020), it is entirely unknown in *S. argenteus* until now. We describe the first report of a methicillin-resistant *S. argenteus* that exhibited HmupR.

## Materials and Methods

### Identification of the Methicillin-Resistant, Mupirocin-Resistant *S. argenteus*

The isolate (1801221) was obtained in April 2018 from a human nasal swab and was previously identified as methicillin-resistant *S. aureus* (MRSA) with HmupR. For characterization, it was sent to the National Reference Center for Staphylococci and Enterococci, Robert Koch Institute, Germany. To delineate *S. argenteus* from *S. aureus*, PCR amplification of the NRPS gene (Becker et al., 2019) was performed at the Institute of Medical Microbiology, Münster. The isolate was subjected to antibiotic susceptibility testing (Vitek 2 automated system bioMérieux, Marcy l’Étoile, France). The minimum inhibitory concentration (MIC) to mupirocin was also determined using the gradient diffusion method (E-test, bioMérieux, Marcy l’Étoile, France). Methicillin and mupirocin resistance was confirmed by PCR detection of *mecA* (Murakami et al., 1991) and *mupA* (Nagant et al., 2016). We interpreted the results of the antibiotic susceptibility testing and E-test according to the EUCAST clinical breakpoints (Version 11.0).

### Whole-Genome Sequencing

The *S. argenteus* isolate was further processed for whole-genome sequencing (WGS) on a Sequel II platform (Pacific Biosciences Inc., Menlo Park, CA, USA). Before sequencing, we constructed the sequence library using the SMRTbell Express Template Prep Kit 2.0 (Pacific Biosciences Inc.) according to the manufacturer’s recommendations. The resulting long-read sequencing data were assembled applying the “Microbial Assembly” pipeline within the SMRT Link software version 9 (Pacific Biosciences Inc.) using default parameters except for the genome size, which was adopted to 2.8 Mb. Then, we utilized the Ridom SeqSphere^+^ software (version 7, Ridom GmbH, Münster, Germany) to *in silico* predict the antimicrobial resistance and virulence genes and to extract the staphylococcal protein A (*spa*) type and the multilocus sequence type (ST) of the isolate. Also, we used the Plasmid Finder (version 2.1) to identify the replicon sequences (Carattoli et al., 2014). Further analysis, and annotation of the sequences, was performed using the NCBI Prokaryotic Genome Annotation Pipeline software revision 5.3 (Tatusova et al., 2016). A Neighbor-Joining (NJ) tree was constructed using sequences of a global collection of 111 *S. argenteus* (ST2250) isolates. Single nucleotide polymorphisms (SNPs) were extracted from 1,864 core genome genes (Leopold et al., 2014) present in all isolates. The SNPs analysis formed the basis to calculate the NJ tree with default parameters within the Ridom SeqSphere^+^ software version 7.

## Results and Discussion

**Figure 1:**
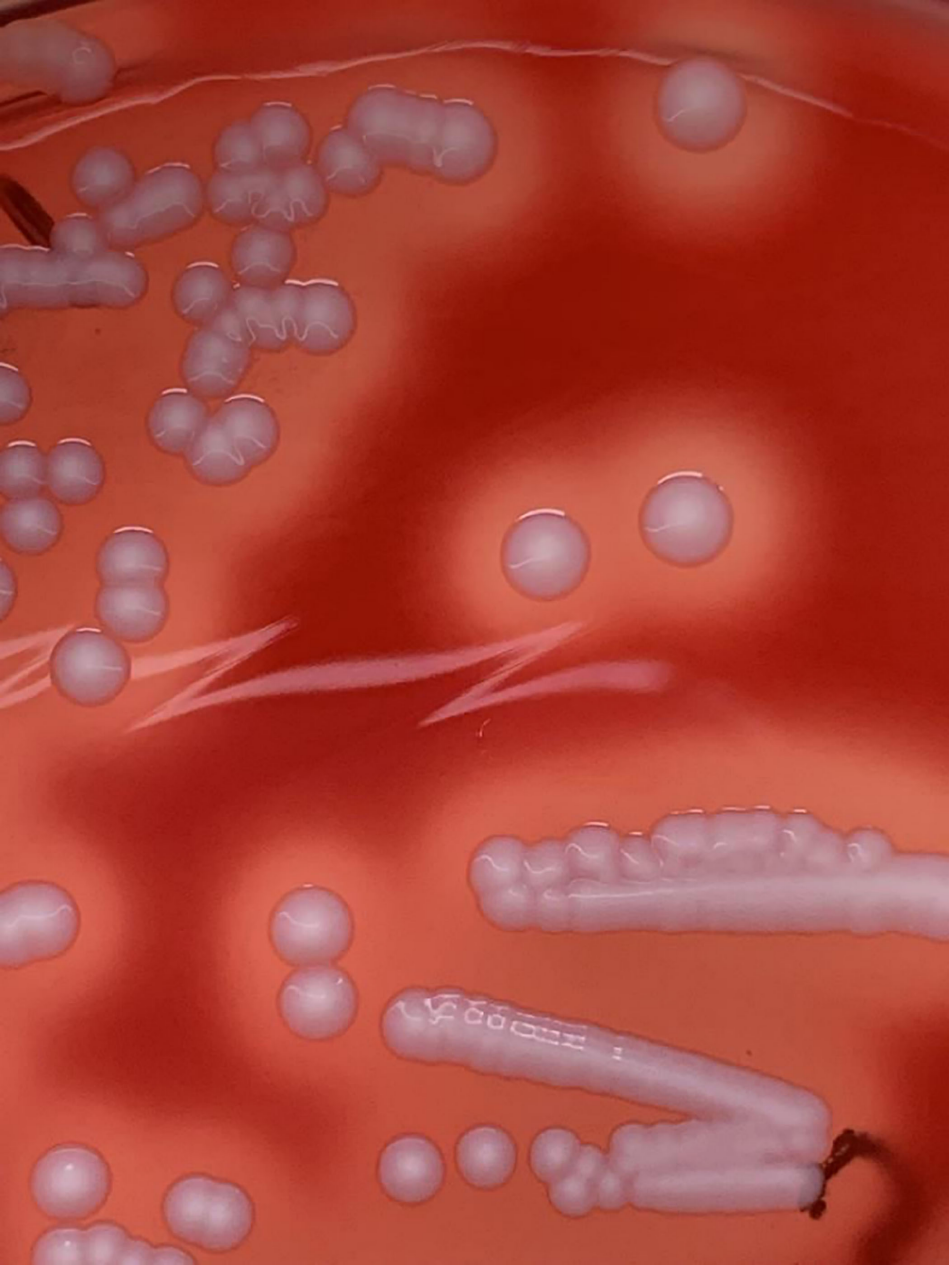
*S. argenteus* (1801221) on Columbia Blood Agar (37°C, 48 hours).

The isolate displayed creamy-white colonies with β-haemolysis on Columbia sheep blood agar (CBA, BD, Heidelberg, Germany) (**Figure 1**). MALDI-TOF identification using the MBT compass (Version 9) did not distinguish reliably between *S. aureus* (Score: 2.04) and *S. argenteus* (Score: 2.13). However, it was PCR-positive (360bp) for the NRPS gene, indicating that it is *S. argenteus*. Antibiotic susceptibility testing showed that the isolate was resistant to cefoxitin, fosfomycin, mupirocin, and trimethoprim/sulfamethoxazole. The MIC of mupirocin (≥512 μg/ml, E-test) was in agreement with the VITEK result (MIC = ≥512 μg/ml). PCR revealed that the isolate was *mecA* and *mupA*-positive. WGS confirmed the identity of the isolate as *S. argenteus* and its antibiotic resistance phenotype. Also, molecular typing characterized the isolate as t6675-ST2250-SCC*mec*IVc. It was associated with capsule type 8, positive for the immune evasion (*sak*, *scn*) gene cluster, haemolysins (*hld*, *hlgB*, *hly*/*hla*), and the intracellular adhesion (*icaA*, *icaB*, *icaC*, *icaD*, *icaR*) gene operon. The isolate was negative for the PVL-encoding gene.

**Figure 2:**
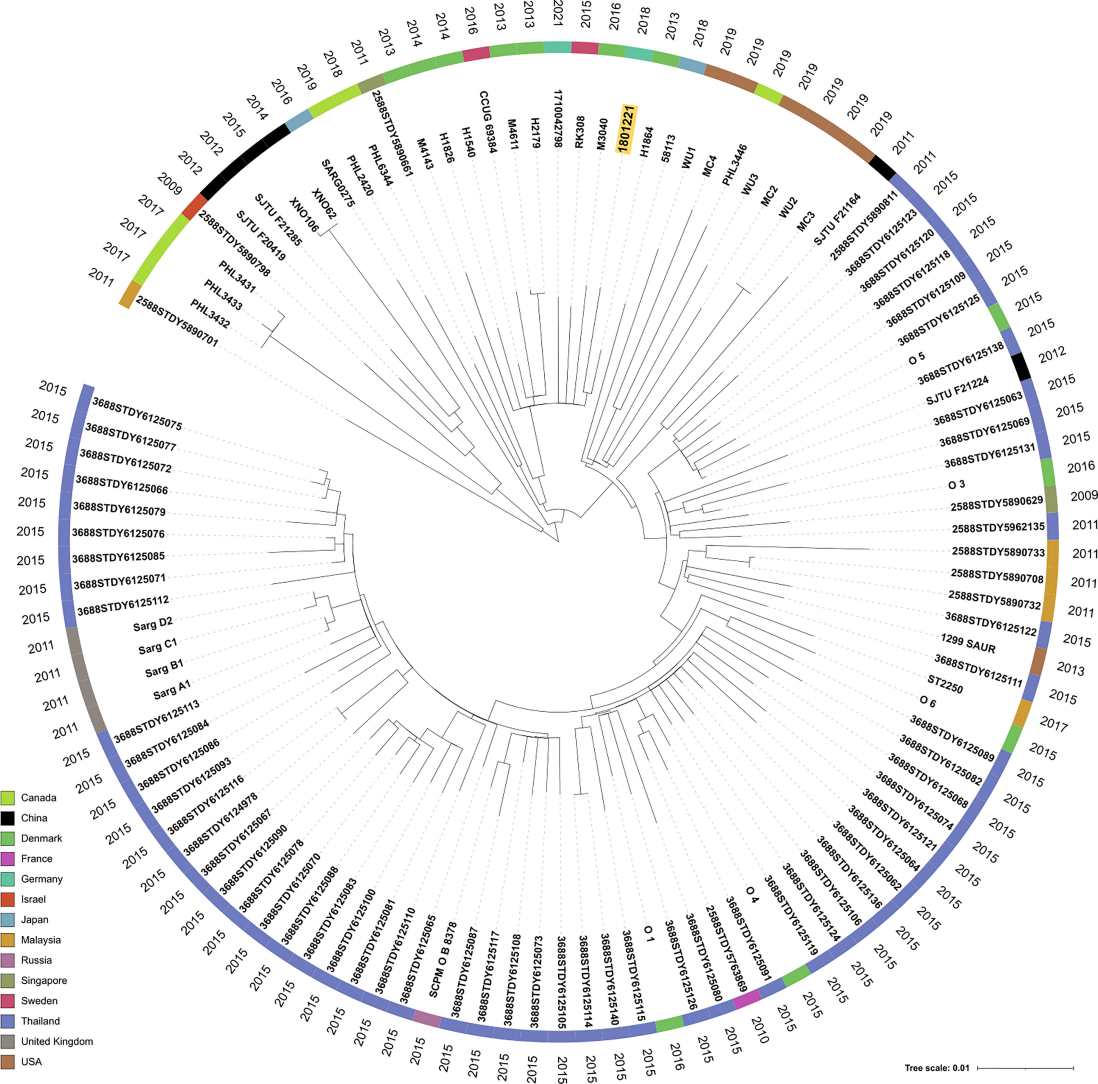
Neighbor-joining (NJ) tree of 111 *S. argenteus* ST2250 global isolates. SNPs (n = 2,177) were extracted from 1,864 core genome genes present in all isolates and formed the basis to calculate the NJ tree with default parameters within the Ridom SeqSphere^+^ software. We used iTOL V. 6 (Letunic and Bork, 2021) to display the tree and metadata of the strains. The leaves of the tree were annotated with the sample names. The colored circle indicates the country of isolation and the outer circle the isolation year, respectively. Isolate 1801221 is highlighted in a yellow box.

**Figure 3:**
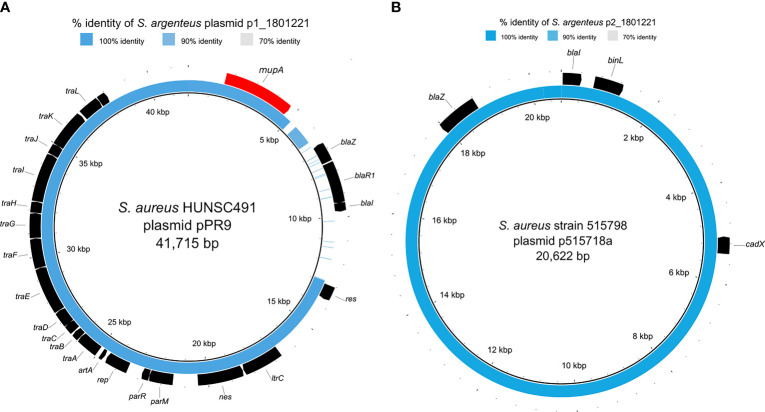
Comparison of *S. argenteus* plasmids with closely related *S. aureus* plasmids. The inner black ring represents the reference sequence, and the blue ring depicts the respective *S. argenteus* plasmid sequence. The outer black ring provides annotation information, i.e., detected ORFs, where the annotation resulted in known genes. The blue color’s intensity is related to the sequence similarity, detailed in the **Supplementary Table**. **(A)** depicts the comparison of p1_1801221 and the conjugative *S. aureus* plasmid pPR9 harboring the *mupA* gene encoding for mupirocin resistance (colored in red); **(B)** shows the comparison of p2_1801221 and the *S. aureus* plasmid p515718a harboring *blaZ* and *cadX* encoding for beta-lactam and cadmium resistance, respectively.

The *S. argenteus* isolate chromosome was 2,781,166 bp in size, with a GC content of 32.3%, containing 2,650 predicted coding DNA sequences (CDSs). The NJ tree based on 2,177 SNPs from a global collection of all available genomes (as of 8 December 2021) of ST2250 *S. argenteus* isolates (**Supplementary Table**) showed that it clustered with the European ST2250 clade (**Figure 2**). The Plasmid Finder identified sequences of two plasmids with replication (*rep5a* [locus tag 13590 in **Supplementary Figure 1B**], *rep16* [locus tag 13610 in **Supplementary Figure 1B**], and *rep23* [locus tag 13385 in **Supplementary Figure 1A**]) genes, respectively. The larger plasmid (34,870 bp), designated p1_1801221, with *rep23* carried *mupA*. This gene demonstrated 100% sequence identity with the alternative isoleucyl-tRNA synthetase (*ileS-2*) gene conferring HmupR on a conjugative plasmid pPR9 from *S. aureus* (GenBank accession number GU237136). Moreover, the whole plasmid was nearly identical at sequence level with the published plasmid pPR9 (**Figure 3A**) using the BRIG tool (Alikhan et al., 2011). The smaller plasmid (20,644 bp), assigned as p2_1801221, with *rep5a* and *rep16*, harboured the penicillin (*blaZ*) and cadmium (*cadD*) resistance genes. Again, the genes and overall plasmid composition exhibited high homology to *S. aureus* resistance determinants and plasmid. Specifically, *blaZ* showed 99.9% sequence identity with the corresponding gene on pN315 (GenBank accession number AP003139), and the *cadD* gene displayed 100% homology with the resistance determinant on pSAS (GeneBank accession number BX571858). Moreover, the plasmid as a whole was nearly identical to the *S. aureus* plasmid p515718a of strain 515798 (GenBank accession number CP045475) (**Figure 3B**).

*S. argenteus* was first reported in northern Australia (McDonald et al., 2006) and distinct from *S. aureus* based on the average nucleotide identity of 87.4% and a DNA-DNA hybridization value of 33.5% (Tong et al., 2015). About 10% of *S. aureus* isolates from human infections are non-pigmented (Zhang et al., 2018). Also, *S. argenteus* colonies on blood agar are non-pigmented (creamy-white) due to the lack of the crt*OPQMN* operon responsible for carotenoid pigment, staphyloxanthin (Holt et al., 2011). Hence, *S. argenteus* and non-pigmented *S. aureus* could be indistinguishable on blood agar based on colony morphology and phenotypic tests (coagulase, DNase). This scenario could be a dilemma in the clinical microbiology laboratory (Becker et al., 2019). This study provided evidence on the reliability of the PCR detection of the NRPS gene with WGS in the delineation of *S. argenteus* from *S. aureus*. ST2250 is a global *S. argenteus* clonal group (Eshaghi et al., 2021), and our first report of an isolate in this clone exhibiting HmupR is of public health importance. MRSA with HmupR is a serious problem as decolonization with mupirocin becomes ineffective (Patel et al., 2009). Moreover, HmupR could facilitate the spread of antibiotic resistance through the conjugative transfer of plasmid mediating HmupR with co-mobilization and co-transfer of plasmids encoding other gene determinants (Udo and Jacob, 1998; Pawa et al., 2000). Also, macrolide, gentamicin, tetracycline, and trimethoprim resistance genes have been identified on the same extra-chromosomal element with *mupA* (McDougal et al., 2010). In this study, the identification and high homology of both plasmids identified in *S. argenteus* with published DNA sequences of *S. aureus* plasmids suggest interspecies transfer.

*S. argenteus* carriage in the human population (Aung et al., 2017; Senok et al., 2020; Eshaghi et al., 2021; Jauneikaite et al., 2021) and possible person-to-person transmission (Giske et al., 2019; Eshaghi et al., 2021) have been described. Moreover, a study revealed that cases of *S. argenteus* bacteremia were associated with higher mortality than methicillin-susceptible *S. aureus* bacteremia (Chen et al., 2018b). *S. argenteus* with different antibiotic resistance genes have been reported (Aung et al., 2021; Eshaghi et al., 2021), including an isolate with elevated MIC (4µg/ml) to daptomycin and vancomycin in the United States (Hao et al., 2020). Recent studies from China (Chen and Wu, 2020) and Japan (Wakabayashi et al., 2021) have also identified *S. argenteus* from retail foods and an emerging bovine mastitis pathogen in Thailand (Pumipuntu, 2019). We could not ascertain if the study individual received mupirocin or not. Nonetheless, these increasing reports and the capacity of *S. argenteus* to harbor resistance gene determinants (including *mupA*) with its repertoire of virulence factors highlight the need for its delineation from *S. aureus* and correct identification. Therefore, enhanced surveillance is vital to understanding the significance of *S. argenteus* in clinical and non-clinical settings.

## Data Availability Statement

The whole-genome sequence project for the S. argenteus; isolate (1801221) has been deposited in NCBI under the bioproject accession number PRJNA764657 with sequence accession numbers CP083805-CP083807 for the chromosome and the two plasmids.

## Authors Contributions

AS, FL-N, BS, and FS designed the research. AS, M-TN, SB, and AM performed the experiments. AS, SB, and AM analyzed the data. AS wrote the initial draft of the manuscript. All authors contributed to the article and approved the submitted version.

## Funding

This study received support from the Deutsche Forschungsgemeinschaft (SCHA 1994/5-1, granted to AS and FS) and the Alexander von Humboldt Foundation (“Georg Forster-Forschungsstipendium” granted to AS). We acknowledge support from the Open Access Publication Fund of the University of Muenster.

## Conflict of Interest

The authors declare that the research was conducted in the absence of any commercial or financial relationships that could be construed as a potential conflict of interest.

## Publisher’s Note

All claims expressed in this article are solely those of the authors and do not necessarily represent those of their affiliated organizations, or those of the publisher, the editors and the reviewers. Any product that may be evaluated in this article, or claim that may be made by its manufacturer, is not guaranteed or endorsed by the publisher.

## Supplementary Material

Supplementary Figures 1a and 1b Circular illustration of the two *S. argenteus* plasmids and annotation of antibiotic resistance genes.
