# Supplementary figures and images for: First Report of a Methicillin-Resistant, High-Level Mupirocin-Resistant Staphylococcus argenteus
Source: Front Cell Infect Microbiol. 2022 Mar 15;12:860163. doi: 10.3389/fcimb.2022.860163 (PMC8964999; doi:10.3389/fcimb.2022.860163)

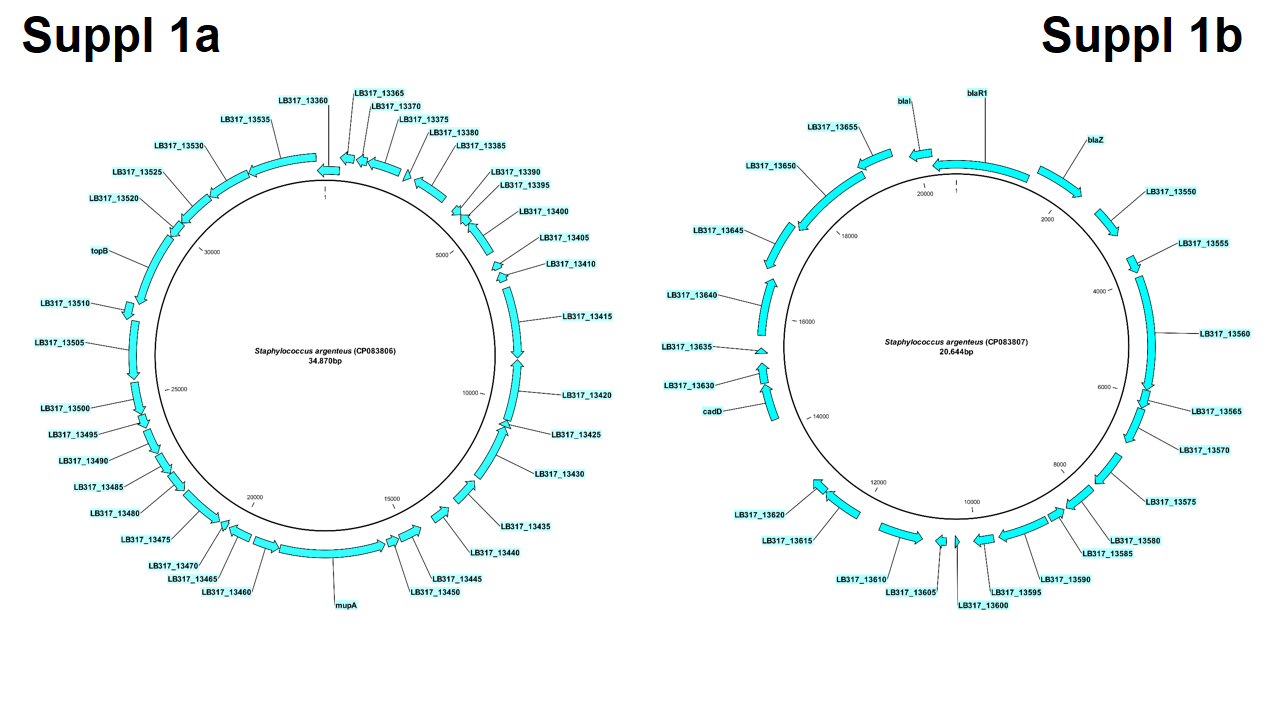

Supplement: Supplementary Figures 1a and 1b — Circular illustration of the two S. argenteus plasmids and annotation of antibiotic resistance genes. [file Image_1.tif]
